# Interval Outcomes of a Lifestyle Weight-Loss Intervention in Early Adolescence

**DOI:** 10.3390/children5060077

**Published:** 2018-06-15

**Authors:** Christina Wei, Toby Candler, Elizabeth Crowne, Julian P. Hamilton-Shield

**Affiliations:** 1 Department of Paediatric Endocrinology & Diabetes, Bristol Royal Hospital for Children, University Hospitals Bristol NHS Foundation Trust, Upper Maudlin Street, Bristol BS2 8BJ, UK; christina.wei@nhs.net (C.W.); toby.candler@UHBristol.nhs.uk (T.C.); Liz.Crowne@UHBristol.nhs.uk (E.C.); 2 Department of Paediatric Endocrinology, St George’s University Hospitals NHS Foundation Trust, Blackshaw Road, London SW17 0QT, UK; 3 NIHR Bristol Biomedical Research Centre, Nutrition Theme, University of Bristol, UHBT Research and Education Building, Upper Maudlin Street, Bristol BS2 8AE, UK

**Keywords:** adolescents, obesity, weight management

## Abstract

We undertook a feasibility study to reassess metabolic outcomes in young people with early onset obesity who attended a hospital-based lifestyle weight-loss intervention during adolescence. Comparisons of metabolic assessments, including body mass index standard deviation scores (BMI–SDSs), blood pressure (BP), oral glucose tolerance tests (OGTTs), lipid profile, and alanine transaminase (ALT), before and after treatment were made. Twenty-five subjects (10 males) with median ages (interquartile range, IQR) of 14.5 (12.6–15.4) years at the beginning of intervention and 18.2 (17.2–18.9) years at reassessment and who were 3.5 (2.4–6.5) years post-intervention were recruited. Twenty-eight percent had a ≥0.25 reduction in BMI–SDS from baseline (responders). Responders demonstrated significantly lower BMI–SDS, systolic BP, and glucose disposal at reassessment compared with baseline. They also showed significantly lower total fat percentage SDSs, trunk fat percentages, 120 min insulin, and ALT, as well as higher insulin sensitivity index (ISI_comp_) than non-responders. Male gender and younger age at the initiation of intervention showed a non-significant trend towards greater success in weight loss. Long-term benefits were demonstrated in around one-quarter of obese adolescents after lifestyle modification treatment, with associated improvements in body composition and metabolic parameters.

## 1. Introduction

Obesity increases the risk of many long-term health conditions, such as early onset type 2 diabetes mellitus, atherosclerotic cardiovascular disease, stroke, degenerative arthritis, obstructive sleep apnea, non-alcoholic fatty liver disease, infertility, and various cancers (e.g., breast and bowel) [1]. Furthermore, obesity in women of childbearing age pre-pregnancy and during pregnancy results in heritable risk factors for cardiometabolic disease in offspring, possibly by programming [2]. Obesity management costs escalate over time with the increased manifestation of comorbidities. Therefore, it is important to target individuals with obesity as early as possible in life.

Lifestyle and behavioural interventions based on modification of diet and physical activity are recommended as the first-line treatment for obesity during childhood and adolescence [3]. Systematic reviews have demonstrated some short-term success after such interventions [4]. A minimum of a 0.25 reduction in the body mass index standard deviation score (BMI–SDS) has been shown to improve adiposity and metabolic health in children [5]. Unfortunately, most children with obesity will become obese adults [6], putting them at risk of significant obesity-related morbidity, such as type 2 diabetes, hypertension, hyperlipidaemia, and non-alcoholic fatty liver disease. Longer-term information on the maintenance of BMI–SDS loss and its health implications for those undergoing interventions for obesity during childhood is lacking. We undertook a feasibility study to investigate the metabolic outcomes of young people who received lifestyle interventions during adolescence for early onset obesity.

## 2. Materials and Method

All subjects gave their informed consent for inclusion before they participated in the study. The study was conducted in accordance with the Declaration of Helsinki and was approved by the National Research Ethics Committee South West–Central, Bristol, UK (Medical Research Ethics Committee No. 10/H0102/67). We aimed to recruit 25–30 young adults aged 16–25 years for metabolic reassessments with childhood-onset obesity who had previously undergone a lifestyle weight-loss intervention during adolescence in a children’s hospital clinic. All had a BMI of ≥98th centile at the beginning of treatment. Referrals were from community health professionals: health visitors, school nurses, and general practitioners. The multidisciplinary team consisted of a paediatrician, a dietician, and an exercise specialist, with access to psychological services if clinically indicated. Management included an initial general health assessment and metabolic screening for obesity-related comorbidity followed by four-monthly follow up for a minimum of one year. At each clinic visit, all subjects underwent anthropometry and received behavioural modification advice for their diet (e.g., healthy options, appropriate calorific intake, and portion size) and exercise habits (e.g., type and amount per day). The subjects for the present study were identified from the department’s patient database and were approached in reverse chronological order from the last clinic appointment date. All female subjects underwent a pregnancy test on the assessment day. Informed consent was obtained from all participants.

Demographic data was collected in clinic. Weight, height, and BMI (weight (kg) divided by height squared (m^2^)) were measured and converted to SDSs according to UK 1990 reference data (Medical Research Council, Swindon, UK) [7]. Metabolic screening included the following: (1) blood pressure (BP), measured by an automatic oscillometric method (Dinamap Critikon 8100; Critikon, Tampa, FL, USA); (2) total body fat, trunk body fat, and fat-free mass percentages (%), measured by bioimpedance (BIA) using a Tanita bioimpedance segmental body composition analyser (model BC-418MA; Tanita, Arlington Heights, IL, USA). Total body fat % was converted to fat %–SDS as per population references [7]. Bioimpedance measures body resistance to electrical current (i.e., the electrical impedance) at a defined frequency through body tissues to calculate an estimate of total body water. Fat-free body mass is derived from total body water on the assumption that 73% of the body’s fat-free mass is water. Body fat is calculated from the differences between fat-free mass and body weight [8]. Trunk fat % is defined as the total fat % minus fat % in all four limbs. (3) Blood tests included a fasting lipid profile measurement (total cholesterol (TC), high-density lipoprotein (HDL), and triglycerides (TG)), alanine transaminase (ALT) measurement, and oral glucose tolerance tests (OGTTs). The OGTT protocol involved an 8–12 h overnight fast with venous glucose levels taken at baseline and 30, 60, 90, and 120 min following ingestion of oral glucose solution (1.75 g/kg; maximum of 75 g). Total cholesterol, HDL, and TG were measured by enzymatic colorimetric assays, glucose by hexokinase methods, and ALT spectophotometrically (COBAS analyser, Roche Professional Diagnostics Products, Welwyn Garden City, UK). Insulin sensitivity was defined by the whole-body composite insulin sensitivity index (ISI_comp_) according to the following formula: 10,000 ÷ $\sqrt{\mathrm{fasting}\;\mathrm{glucose}\;\times \;\mathrm{fasting}\;\mathrm{insulin}\;\times \;\mathrm{mean}\;\mathrm{glucose}\;\times \;\mathrm{mean}\;\mathrm{insulin}}$ [9].

We recorded dates of the first and last attended appointments as well as the total number of clinic appointments offered and attended by the patients during intervention. Compliance with intervention was defined as the number of attended appointments divided by the total number of appointments offered over the duration of intervention.

Results were compared by Mann–Whitney *U* tests, Fisher’s exact tests, and Spearman’s ρ non-parametric correlation using SPSS Statistical Software version 23 (IMB, Armonk, NY, USA) at 5% significance with results reported in medians and interquartile ranges (IQRs).

## 3. Results

### 3.1. Demographics

The 133 eligible subjects from the clinical database were approached, among which 65 were non-contactable, 31 did not wish to take part, 7 withdrew after having initially agreed to take part, and 5 cases were excluded as they received weight-loss interventions for under one year, resulting in a total of 25 cases (10 males) for the analysis of metabolic outcomes, representing 18.7% of the clinical cohort. No female participants were pregnant. There were 21 Caucasian, two South Asian, and two mixed Caucasian/Black participants of median (IQR) ages of 14.5 (12.6–15.4) years at the beginning of intervention and 18.2 (17.2–18.9) years at reassessment. Subjects were 3.5 (2.4–6.5) years post-intervention at the time of metabolic assessment in the study. Body mass index standard deviation scores reduction was greater than 0.25 from baseline in 28% (7/25) of the subjects (referred to as responders in the rest of this report).

### 3.2. Metabolic Outcomes

All results are listed in Table 1. In summary, responders demonstrated a significant reduction in BMI–SDS, systolic BP, and glucose area under the curve from OGTT, as well as an increase in fat-free mass % at reassessment compared with baseline. Non-responders showed significant increases in total fat %–SDS and trunk fat %. There were no differences between responders and non-responders in terms of metabolic outcome at baseline before the intervention. At reassessment, responders compared with non-responders showed significantly lower BMI–SDS, fat %–SDS, trunk fat %, insulin at 120 min from OGTT, and ALT, as well as higher ISI_comp_, but there were no group differences in diastolic BP, TG, and HDL or fat free mass %. There were no patients with impaired glucose tolerance or diabetes in either group at baseline or reassessment.

### 3.3. Potential Factors Associated with Maintenance of BMI–SDS Reduction

More males (5/10) than females (2/15) maintained a >0.25 BMI–SDS reduction, although the result was not statistically significant (*p* = 0.075). There were no statistical differences in pubertal status, BMI–SDS at the start of intervention, or social deprivation scores between the responders and non-responders. There was a trend towards greater BMI reduction in subjects treated at a younger age, but again this did not reach statistical significance (*r* = −0.37, *p* = 0.07).

There were no differences between the responders and non-responders in terms of the length of intervention offered (1.82 (1.3–4.87) years vs. 2.44 (1.97–4.94) years; *p* = 0.36) or compliance with the intervention (0.75 (0.54–0.9) years vs. 0.75 (0.59–1.0) years; *p* = 0.91).

## 4. Discussion

Our study suggested that slightly over one in four obese adolescents may benefit in the longer term after lifestyle modification interventions with associated improvements in body composition and metabolic parameters. Although the BMI–SDSs of all subjects in this cohort remained in the obese range at reassessment, those who achieved and maintained weight loss after intervention still benefitted from improved metabolic outcomes three to four years later. This message may motivate some obese individuals who will realistically never achieve an ideal BMI.

Type 2 diabetes is the most common complication associated with obesity and is characterised by reduced insulin sensitivity and increased visceral fat. Our results showed significantly lower trunk fat % and improved insulin sensitivity, as indicated by lower stimulated insulin levels and higher ISI_comp_ values in subjects who maintained weight loss. Responders also showed a significantly lower ALT, a marker of non-alcoholic fatty liver disease. Non-alcoholic fatty liver disease is another well-known comorbidity of obesity that may lead to liver cirrhosis. Previous data have shown that multidisciplinary lifestyle interventions resulting in a reduction in BMI–SDS improve transaminitis in children and adolescents with obesity [10]. However, longer-term follow-up with a greater number of subjects is needed to show whether these markers translate to a significant risk reduction in the development of type 2 diabetes and non-alcoholic fatty liver disease in later life.

As a feasibility study, the number of subjects was too small and underpowered to demonstrate statistical significance. The low participation percentage in this study may be a real-life reflection on the challenges of engaging individuals in lifestyle changes to enable fat loss and maintenance of a healthy weight. There were possible trends indicating greater success in maintenance of weight loss among male subjects and those referred for intervention at a younger age. Interestingly, these factors were associated with greater success in a different cohort from the same obesity clinic [11]. Body composition changes during puberty, and females acquire greater body fat % than males. However, the improvements in metabolic parameters among the responders were not likely to be only a reflection of the male dominance among the responders, as outcomes of the body fat measurements were corrected for age and gender, with outcomes converted to SDS scores before analysis. However, results of trunk fat % and fat-free mass % were not converted to SDS because of the absence of local reference data. Future studies with a population control group or using alternative methods such as dual energy X-ray absorptiometry (DEXA) with available age-corrected local reference data will address this limitation.

Perhaps surprisingly, the maintenance of BMI–SDS reduction was not related to the length of attendance or compliance to the intervention. In clinical practice, this means that patients resistant to weight loss are unlikely to succeed over a longer period of attendance. This may enable clinicians to justify discharging patients after a limited period of follow up regardless of the outcome for weight reduction. Further studies should evaluate the cost-effectiveness of weight-loss interventions by identifying factors that influence success and failure in order to inform screening criteria for entry into specialised clinics as well as to explore the optimal amount of weight loss and its longer-term benefits in later adulthood.

In summary, we demonstrate positive effects on metabolic health in early adulthood for individuals with early onset obesity who maintained BMI–SDS changes after a lifestyle weight intervention during early adolescence. Whilst by no means ubiquitous in effect, a simple intervention that improves the anthropometry and metabolic health in the longer term of one in four obese adolescents undergoing therapy is relatively encouraging, given the pandemic of adult obesity. Further studies are needed to assess the overall cost-effectiveness of weight-loss interventions implemented for obese children.

## Author Contributions

C.W. was involved in the study design, patient recruitment, conduction of clinical investigations, data collection, and statistical analysis and wrote the first draft of the manuscript. T.C. assisted in data collection and contributed to the writing of the manuscript. E.C. and J.P.H.-S. were involved in the study design and writing of the manuscript and had overall responsibility in the supervision of the study.

## Figures and Tables

**Table 1:** Metabolic markers of responders baseline vs. reassessment responders vs. non-responders at reassessment.

	Responders at Baseline (*n* = 7) Median (IQR)	Responders at Reassessment (*n* = 7) Median (IQR)	Responders’ Baseline vs. Reassessment *p*-Value	Non-Responders at Baseline(*n* = 18) Median (IQR)	Non-Responders at Reassessment (*n* = 18) Median (IQR)	Non-Responders’ Baseline vs. Reassessment *p*-Value	Responders vs. Non-Responders at Reassessment *p*-Value
BMI–SDS	3.36 (3.2–3.6)	2.98 (2.5–3.2)	0.017	3.0 (2.5–3.4)	3.34 (3.1–3.8)	0.08	0.034
Systolic BP (mmHg)	136 (133–147)	115 (111–122)	0.007	128 (112–138)	114 (106–134)	0.11	0.70
Diastolic BP (mmHg)	67 (61–66)	68 (37–42)	0.53	64 (51–71)	65 (36–46)	0.37	0.53
Fat %–SDS (%)	2.9 (2.5–3.7)	2.9 (2.8–3.3)	0.56	2.6 (2.3–3.3)	3.4 (3.0–3.6)	0.003	0.008
Glucose: 0 min at OGTT (mmol/L)	4.6 (4.4–5.0)	4.4 (4.2–7.0)	0.53	4.5 (4.4–4.8)	4.7 (4.4–5.0)	0.85	0.09
Glucose: 120 min at OGTT (mmol/L)	6.3 (5.8–6.8)	3.7 (3.4–6.7)	0.14	6.0 (5.8–6.8)	5.3 (4.4–7.2)	0.12	0.11
Insulin: 0 min at OGTT (mU/L)	18.0 (11.2–21.6)	14.3 (7.4–18.6)	0.53	17.5 (4.7–25.3)	24.5 (13.4–35.2)	0.13	0.09
Insulin: 120 min at OGTT (mU/L)	78 (51.3–123.9)	16 (6.7–75.6)	0.18	95.8 (65.8–145.5)	92.4 (25.3–180.8)	0.80	0.029
Glucose: AUC at OGTT	14.2 (13.1–15.3)	11.7 (9.9–13.1)	0.008	13.2 (12.0–14.4)	13.0 (11.2–14.8)	0.96	0.06
ISI_comp_	NA	3.81 (2.28–5.29)	NA	NA	1.73 (0.96–3.59)	NA	0.021
Total cholesterol (mmol/L)	4.5 (3.0–4.6)	4.5 (3.9–4.8)	0.64	4.4 (4.0–5.1)	4.8 (4.1–5.3)	0.27	0.20
HDL (mmol/L)	1.1 (0.9–1.2)	1.1 (1.1–1.2)	0.43	1.2 (1.0–1.3)	1.2 (1.0–1.3)	0.83	0.84
TG (mmol/L)	1.1 (1.1–1.7)	1.3 (1.1–1.4)	0.88	1.4 (1.1–1.7)	1.4 (1.0–1.4)	0.73	0.98
ALT (U/L)	24 (18.0–24.0)	26 (19.0–32.0)	1.0	30.5 (22.3–45.8)	34 (25.8–46.8)	0.47	0.041

NA: not available; IQR: interquartile range; BMI–SDS: body mass index standard deviation scores; BP: blood pressure; %: percentage of; OGTT: oral glucose tolerance test; AUC: area under the curve; ISI_comp_: insulin sensitivity index, HDL: high density lipoprotein; TG: triglycerides; ALT: alanine transaminase.

## References

[1] Kelsey M.M., Zaepfel A., Bjornstad P., Nadeau K.J. (2014). Age-related consequences of childhood obesity. Gerontology.

[2] Roberts V.H., Frias A.E., Grove K.L. (2015). Impact of maternal obesity on fetal programming of cardiovascular disease. Physiology.

[3] NICE guidelines (2014). Obesity: Identification, Assessment and Management.

[4] Whitlock E.P., O’Connor E.A., Williams S.B., Beil T.L., Lutz K.W. (2010). Effectiveness of weight management interventions in children: A targeted systematic review for the USPSTF. Pediatrics.

[5] Ford A.L., Hunt L.P., Cooper A., Shield J.P. (2010). What reduction in BMI–SDS is required in obese adolescents to improve body composition and cardiometabolic health?. Arch. Dis. Child..

[6] Gordon-Larsen P., The N.S., Adair L.S. (2010). Longitudinal trends in obesity in the United States from adolescence to the third decade of life. Obesity.

[7] Pan P., Cole T. (1990). UK 1990 IMS Growth Reference.

[8] Kyle U.G., Bosaeus I., De Lorenzo A.D., Deurenberg P., Elia M., Gomez J.M., Heitmann B.L., Kent-Smith L., Melchior J.C., Pirlich M. (2004). Composition of the, E. W. G.; Bioelectrical impedance analysis—Part I: Review of principles and methods. Clin. Nutr..

[9] Matsuda M., DeFronzo R.A. (1999). Insulin sensitivity indices obtained from oral glucose tolerance testing: Comparison with the euglycemic insulin clamp. Diabetes Care.

[10] Wei C., Ford A., Hunt L., Crowne E.C., Shield J.P. (2011). Abnormal liver function in children with metabolic syndrome from a UK-based obesity clinic. Arch. Dis. Child..

[11] Sabin M.A., Ford A., Hunt L., Jamal R., Crowne E.C., Shield J.P. (2007). Which factors are associated with a successful outcome in a weight management programme for obese children?. J. Eval. Clin. Pract..

